# Maternal and Neonatal Hair Cortisol Levels Are Associated with Infant Neurodevelopment at Six Months of Age

**DOI:** 10.3390/jcm8112015

**Published:** 2019-11-19

**Authors:** Rafael A. Caparros-Gonzalez, Borja Romero-Gonzalez, Raquel Gonzalez-Perez, Lidia Lucena-Prieto, Miguel Perez-Garcia, Francisco Cruz-Quintana, Maria Isabel Peralta-Ramirez

**Affiliations:** 1 Department of Nursing, Faculty of Health Sciences, University of Granada, 18071 Granada, Spain; 2 Mind, Brain, and Behavior Research Center (CIMCYC), University of Granada, 18011 Granada, Spain; mperezg@ugr.es (M.P.-G.); fcruz@ugr.es (F.C.-Q.); mperalta@ugr.es (M.I.P.-R.); 3 Personality, Assessment and Psychological Treatment Department, University of Granada, 18071 Granada, Spain; 4 Department of Pharmacology, CIBERehd, Instituto de Investigacion Biosanitaria ibs.GRANADA, School of Pharmacy, University of Granada, 18011 Granada, Spain; raquel.gonzalez@ciberehd.org; 5 Department of Obstetrics & Gynecology, Delivery Ward, Hospital of Antequera, Antequera, 29200 Malaga, Spain; lidilla81@gmail.com

**Keywords:** pregnancy, cortisol, stress, infant, neurodevelopment

## Abstract

Background: Maternal stress during pregnancy can affect fetal development during certain sensitive periods. Objective: To longitudinally assess maternal hair cortisol levels during pregnancy, and the postpartum along with neonatal hair cortisol levels that could be associated with infant neurodevelopment at six months of age. Methods: A sample of 41 pregnant women longitudinally assessed during the first, second, and third trimester and the postpartum, along with their 41 full-term neonates participated in this study. Hair cortisol levels were assessed from participants. Infant neurodevelopment was assessed by means of the Bayley Scale of Infants Development, Third Edition at age six months. Results: Maternal hair cortisol levels in the first and second trimester accounted for 24% and 23%, respectively, of variance of infant gross motor development (*p* < 0.05). Maternal hair cortisol levels during the postpartum accounted for 31% of variance of infant cognitive development (*p* < 0.05), and 25% of variance of infant gross motor development (*p* < 0.05). Neonatal hair cortisol levels accounted for 28% of variance of infant gross motor development (*p* < 0.05). Conclusions: The preconception and prenatal time are sensitive periods related to infant neurodevelopment along with the cortisol levels surrounding the fetus while in the womb. Pregnant women could be assessed for hair cortisol levels while attending a prenatal appointment.

## 1. Introduction

Exposure to high antenatal stress increases the risk of developing behavioral, emotional, and cognitive problems in later life [1,2,3]. Maternal stress during pregnancy affects fetal development during certain sensitive periods that can result in potentially permanent changes and lifelong consequences for the health of infants [4].

The underlying mechanism responsible for the transfer of maternal stress to the fetus involves maternal cortisol crossing the placenta to the fetal blood circulation [5]. During pregnancy, the placenta, an endocrine organ of fetal origin, alters the hypothalamic–pituitary–adrenal axis [6]. Through an intense increase of placental corticotropin-releasing hormone (CRH) during gestation, the placenta increases the release of cortisol from the adrenal gland [7]. Additionally, high cortisol levels during pregnancy also result from the pituitary glands increasing in size and the positive effect cortisol has on increasing placental CRH production [8].

Cortisol levels have previously been assessed from blood, urine, saliva, or amniotic fluid samples in pregnant women [9,10,11]. These matrixes inform on only acute stress levels, require invasive and sometimes painful techniques, and are influenced by circadian rhythms and environmental variables [8,12]. Testing cortisol levels from hair samples is an advanced technique that informs of chronic stress of the last three months; is not affected by variables in the context such as noise, temperature, or social interaction; is not invasive; and is simple to transport and conserve [13,14,15].

The development of the fetal brain can be altered as a consequence of exposure to high levels of cortisol [16]. Among the factors associated with child neurodevelopment (e.g., maternal smoking, exposure to heavy metals), maternal stress during pregnancy may be a crucial determinant of delay in cognitive and motor development among infants [17]. Nevertheless, not all studies have reported such connections [18]. In this respect, an association between high cortisol levels during pregnancy and high motor and cognitive infant development has been reported [19]. Other studies have stated that low levels of cortisol may predict an accelerated cognitive development during the postpartum and the first 12 months of age [9,20,21].

Since cerebral development of the fetus goes through different stages [22], it is probable that cortisol can alter or stimulate brain development depending on the phase in which it is [23,24].

The aim of this study was to assess maternal hair cortisol levels during the first, second, and third trimester, along with neonatal hair cortisol levels that could predict infant neurodevelopment. We hypothesized that cortisol levels assessed from hair samples affect infant neurodevelopment differently depending on the trimester of pregnancy evaluated.

## 2. Methods

### 2.1. Participants

All subjects gave their informed consent for inclusion before they participated in the study. The study was conducted in accordance with the Declaration of Helsinki (World Medical Association, 2008) and the Good Clinical Practice Directive (Directive 2005/28/EC) of the European Union. The protocol was approved by Human Ethics Research Committee of the University of Granada (reference code 881).

Eligible participants were low-risk pregnant women in the first trimester, proficient in the Spanish language, over 18 years old, and not on steroid medication.

**Figure 1:**
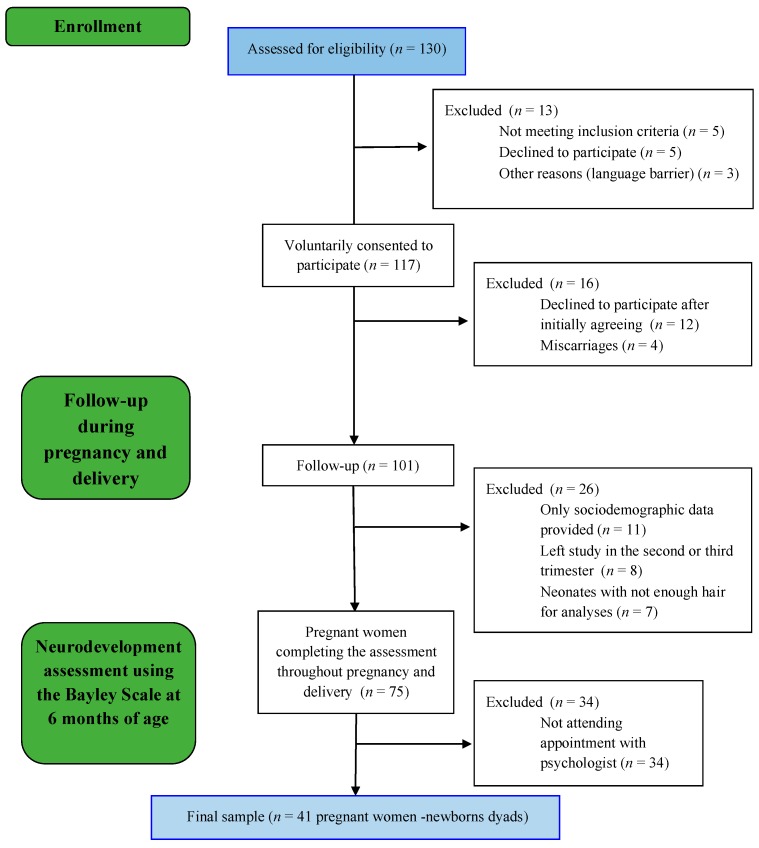
Participants’ follow-up flow diagram.

A total of 130 pregnant women were invited to participate in this study (Figure 1). Due to a variety of reasons (e.g., some of the invited participants not meeting inclusion criteria, pregnancy ending in miscarriage, neonates not having enough hair to be assessed), the final study sample was composed of 41 pregnant women and their 41 neonates. In order to obtain satisfactory results, a priori sample size requirements according to G*Power Statistical software was established at a minimum sample size of 34 to acquire an adequate power of 0.8 and using an α = 0.05. Infants in this study were stable at birth with 1 min Apgar score ranging from 9 to 10 (M = 9.5, SD = 0.5). Women were included in the final sample if they completed all assessment points.

### 2.2. Procedure

Maternal hair samples were evaluated at three points in time during pregnancy, coinciding with a prenatal appointment with a community midwife (Time 1: 12 weeks of gestation ± 3.31, Time 2: 24 weeks of gestation ± 3.09, Time 3: 34 weeks of gestation ± 2.69). After birth, maternal and newborn hair samples were assessed at one point in time, coinciding with a postnatal appointment with a community midwife (Time 4: 10 days after birth ± 2.4). Six months after birth, a trained psychologist assessed the infant’s neurodevelopment at our laboratory at the university (Time 5: 6.25 months ± 0.39). The assessment of each infant’s neurodevelopment took between 40 and 50 min. Participants received 10 euros as a compensation in order to cover travel expenses and a report informing the level of neurodevelopment of their infants.

Additionally, medical, obstetric, and sociodemographic information was obtained from the official pregnancy health document.

### 2.3. Predictor Variables

#### Maternal, Newborn, and Infant Hair Cortisol Assay

Hair cortisol levels were obtained from hair samples proximal to the scalp with a maximum length of 3 cm (an average growth rate of 1 cm/month was assumed; a 3 cm segment contains cortisol deposited over the last three months). Each sample was collected from the posterior vertex of the head and consisted of approximately 150 strands of hair [25]. Then, the hair samples were wrapped in aluminum foil, stored in an envelope at room temperature, and sent for analysis to the collaborative laboratory. After incubating the samples for 72 h at room temperature in the dark with constant inversion using a rotator, cortisol from the hair samples was extracted into high-performance liquid chromatography (HPLC)-grade methanol. At that time, the supernatant was dried using a vacuum evaporator (Centrivac, Heraeus, Hanau, Germany) and the resulting extract was reconstituted in 150 µL of phosphate buffered saline with a pH of 8.0. Afterward, the reconstituted sample was frozen at −20 °C for later analysis [25,26,27].

Following the manufacturer’s directions, the Salivary ELISA Cortisol Kit© was used to measure hair cortisol levels (Alpco Diagnostics^®^, Windham, NH, USA). The ELISA Cortisol Kit is a validated tool to measure hair cortisol levels and is highly positive correlated with liquid chromatograph–mass spectrometry (LC–MS/MS) [28].

Eight consecutive assays measured in duplicate were applied on internal quality controls to check the intra- and inter-assay variations for routine salivary cortisol measurement. The intra-assay coefficients of variance (CV) were 2.7% at 10.7 ng/mL and 4.3% at 43.9 ng/mL. The inter-assay CVs were 4.4% and 6.3%, respectively.

### 2.4. Outcome Variables

#### 2.4.1. Infant Neurodevelopment

We used the Bayley Scales of Infant Development, Third Edition (BSID-III), developed in 2006 by Nancy Bayley, to assess infant neurodevelopment levels. The BSID-III is a standardized assessment tool to measure infant and toddler neurodevelopment from 1 to 42 months. The BSID-III has been successfully used to assess potential links between prenatal stress and infant neurodevelopment [29,30,31].

The BSID-III presents high internal consistency and has been validated in a wide range of countries. Measurements of neurodevelopment were obtained at six months using the cognitive, language, and motor scales. The cognitive scale is comprised of 91 items assessing sensorimotor development, exploration and manipulation, object relatedness, concept formation, and memory. All assessments were performed by the same psychologist at our research laboratory.

#### 2.4.2. Medical, Obstetric, and Sociodemographic Information

Medical and obstetric information included data regarding parity, previous miscarriages, type of delivery, pregnancy method, and medical risk. Sociodemographic information included age, nationality, marital status, employment, salary, and level of education.

Medical, obstetric, and sociodemographic information was obtained from the Official Health Pregnancy Document developed by Junta de Andalucia in 2003.

### 2.5. Statistical Analyses

Because hair cortisol levels did not present a normal distribution, a natural log transformation (natural log; LN base e) was performed.

Maternal level of education and salary have been reported to be associated with infant’s neurodevelopment in previous studies [32,33]. Infant birthweight is a major factor associated with long-term development [5,34]. For this reason, this three factors were included and controlled for in statistical analysis. Considering that maternal age varied from 22 to 39 years old, maternal age was included as a confounding variable in the analyses.

To compare whether there were any differences between those participants in the final sample (Group 1) and those pregnant women who left the study before completing all the assessment points (Group 2), descriptive analyses of cortisol levels were completed. Using a two-sample *t*-test, we compared maternal hair cortisol levels, infants’ hair cortisol levels, infants’ sex, maternal level of education, family salary, and infant birth weight, between both groups.

Next, in order to examine the associations between predictors, predicted variables, and covariates, we performed Pearson’s partial correlations. An absence of high correlation (>0.80) between predictors and a variance inflation factor (VIF) of <1.35 indicated that multicollinearity was not present between predictors. Correlations <0.80 and VIF ≤10 indicates lack of multicollinearity, which makes it easier to assess the importance of an individual predictor in a regression analysis [35].

Finally, in order to assess the effect of maternal and infant hair cortisol levels on infant neurodevelopment, we performed hierarchical linear regressions analyses. The models for the regression analyses for each of the predictors were as follows: First, the three covariate predictors (maternal level of education, salary, infant birthweight) were entered into the model (Step 1), followed by a single predictor (maternal or neonatal hair cortisol levels at one point in time) (Step 2). This procedure was followed to assure obtaining a reliable regression model, respecting the rule of having at least 10 participants per predictor [35]. A lack of multicollinearity was assessed by means of the tolerance statistic, with values below 0.1 indicating serious problems [35].

Analyses were conducted using SPSS v20 (IBM, Armonk, NY, USA) for Mac OSX version 10.12.6. (Apple, Cupertino, CA, USA).

## 3. Results

### 3.1. Descriptive Sample Characteristics

Table 1 shows descriptive analyses of the main sociodemographic, obstetrics, and hair cortisol levels throughout pregnancy.

Pregnant women’s age participating in this study ranged from 22 to 39 (M = 31.90 years; SD = 4.15). Thirty-five women (85.40%) were Spanish, 33 women (80.50%) were working, and 20 women (48.8%) had a monthly salary over 2000 €. In respect to their 41 infants (22 girls and 19 boys), all the infants were full term at the time of birth (M gestational age = 39.32 weeks, SD = 1.12 weeks; M weight = 3200.98 g, SD = 377.06 g).

Descriptive analyses revealed no differences in sociodemographic, obstetric, and hair cortisol levels throughout pregnancy between the final sample (Group 1) and those participants that did not attend the neurodevelopment assessment appointment with the psychologist (Group 2) (Table 1).

No differences were found between boys and girls in respect to hair cortisol levels, cognitive, language, and motor development.

### 3.2. Pearson’s Bivariate Correlations between the Predictors

As shown in Table 2, Pearson’s bivariate correlations between the predictors (maternal and infant hair cortisol levels) were all <0.80, and VIF were all <1.35, indicating a lack of multicollinearity. Thus, maternal and hair cortisol levels were used as predictors in a hierarchical linear regression analyses.

### 3.3. Maternal and Infant Hair Cortisol Levels

Maternal hair cortisol levels increased from the first (M = 303.52 pg/mg; SD = 392.35) to the second trimester (M = 422.64 pg/mg; SD = 712.78). During the third trimester of pregnancy maternal hair cortisol levels decreased compared to the first and second trimester (M = 386.46 pg/mg; SD = 338.93). During the postpartum, maternal hair cortisol levels increased (M = 919.27 pg/mg; SD = 1536.71).

Infants’ hair cortisol levels were higher than maternal hair cortisol levels at any time point during pregnancy or the postpartum period (M = 2747.45 pg/mg; SD = 2209.54).

No significant correlations were found between maternal hair cortisol levels and infant hair cortisol levels (Table S1).

### 3.4. Cognitive, Language, and Motor Deveopment among Infants

The cognitive total scores ranged from 25 to 39 (M = 30.78; SD = 3.58). The receptive language total scores ranged from 7 to 15 (M = 11.02; SD = 1.93) and the expressive language total scores ranged from 5 to 18 (M = 9.32; SD = 2.82). The scores referring to fine motor development ranged from 14 to 28 (M = 20.78; SD 2.96) and the gross motor development scores ranged from 17 to 30 (M = 23.83; SD = 3.27).

### 3.5. Hierarchical Linear Regression Analyses for Maternal Hair Cortisol Levels

The results of the hierarchical regression model for maternal hair cortisol levels throughout pregnancy as predictors, adjusted for potential covariates (maternal level of education, salary, and infant birth weight), and infant neurodevelopment as predicted variables are shown in Table 3. The tolerance statistics between predictors ranged from 0.78 to 0.97, indicating a lack of multicollinearity.

Maternal hair cortisol levels in the first trimester could predict 24% of variance of infant gross motor development (*R*^2^ = 0.24, (F = 2.25, *p* < 0.05), β = −0.18, *p* < 0.05). In the second trimester, maternal hair cortisol levels accounted for 23% of variance of infant gross motor development (*R*^2^ = 0.23, (F = 2.11, *p* < 0.05), β = −0.15, *p* < 0.05). During the postpartum period, maternal hair cortisol levels accounted for 31% of variance of infant cognitive development (*R*^2^ = 0.31 (F = 3.26, *p* < 0.05), β = 0.30, *p* < 0.05), and 25% of variance of infant gross motor development (*R*^2^ = 0.25, (F = 2.33, *p* < 0.05), β = 0.21, *p* < 0.05). Thus, high maternal hair cortisol levels during the first and second trimester predicted low motor development. High maternal hair cortisol levels during the first trimester and the postpartum period predicted a higher cognitive development.

### 3.6. Hierarchical Linear Regression Analyses for Neonatal Hair Cortisol Levels

**Figure 2:**
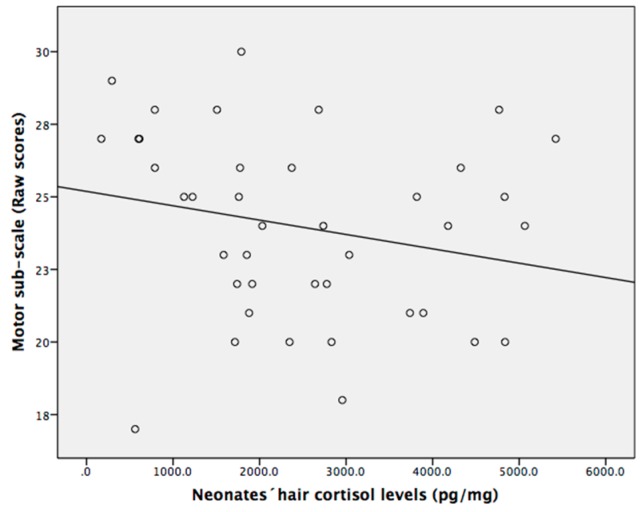
Higher neonatal hair cortisol levels predicted low development in infants. Hair cortisol levels and motor development are raw data.

The hierarchical regression model for infant hair cortisol levels as predictors, adjusted for potential covariates (maternal level of education, salary, and infant birth weight) and infant neurodevelopment as predicted variables are shown in Table 4. The tolerance statistics between predictors ranged from 0.83 to 0.98, indicating a lack of multicollinearity. Hair cortisol levels of newborns predicted 28% of variance of gross motor development in infants (*R*^2^ = 0.28, (F = 2.81, *p* < 0.05) β = −0.31, *p* < 0.05). As shown in Figure 2, high neonatal hair cortisol levels predicted low motor development of infants at six months of age.

A summary of results is provided in Table 5. This table shows how maternal and neonatal hair cortisol levels are associated with infant neurodevelopment depending on the trimester in which cortisol levels were assessed. Table 5 offers the possibility of understanding at a glance the relation between maternal hair cortisol levels at different time points throughout pregnancy and neonatal hair cortisol levels with later infant neurodevelopment at six months of age.

## 4. Discussion

The aim of this longitudinal study was to assess maternal hair cortisol levels during the first, second, third trimester of pregnancy, and the postpartum, along with neonatal hair cortisol levels that may be associated with infant neurodevelopment. We followed a group of pregnant women from the first trimester, through pregnancy, birth, and postpartum until their infants were six months of age. Because maternal level of education, salary, and infant birth weight have been deemed relevant when evaluating infant neurodevelopment [5,32,33], these variables were included as covariates in further analyses. Our results suggested that maternal hair cortisol levels and neonatal hair cortisol levels could predict infant neurodevelopment at the age of six months.

Assessing hair cortisol levels during pregnancy reflects maternal chronic stress during the last three months [15,36]. This measure has been previously used with success in previous studies [6,14]. Biomonitoring neonatal stress levels through hair cortisol levels provides a unique opportunity to assess the level of stress the fetus was exposed to while in the womb [37,38,39]. In our sample, maternal hair cortisol levels ascend from the first trimester to the postpartum. This finding agrees with a previous study reporting hair cortisol levels increased at the end of pregnancy and during the postpartum period among healthy pregnant women [6]. According to our findings, high maternal hair cortisol levels during the first, second, and third trimester were associated with a slower motor development. Our study agrees with those reporting an inverse association between high levels of stress throughout pregnancy and low motor development among infants [17,19,29]. Nevertheless, a previous study using salivary cortisol and a prior edition of the Bayley scale could not find that maternal cortisol during pregnancy predicted low motor development [21]. In our study, during the postpartum period, higher maternal hair cortisol levels predicted an accelerated motor development in infants at six months of age. Our results did not support previous studies that stated high prenatal stress is harmful for the motor development in infants [40]. Due to the fact that hair cortisol levels reflect chronic stress levels during the last three months [41], hair cortisol levels analyzed during the postpartum refer to stress levels the fetus was exposed to in the last trimester.

Our findings regarding hair cortisol levels during the first trimester offer an additional value supporting the importance of assessing stress levels during the periconception period [42]. In this respect, stress levels during the periconception period can have long-term effects on infant development [43].

During postpartum, high hair cortisol levels, which reflected high levels of stress during the third trimester of pregnancy, predicted higher cognitive development in infants [37]. Our findings agree that during the third trimester of pregnancy, levels of placental 11-beta-hydroxysteroid dehydrogenase 2 (11β-HSD2) decrease [44], resulting in higher exposure of fetuses to cortisol. Increasing levels of cortisol at the end of pregnancy are necessary for fetal organ maturation and development [45].

Fetal levels of cortisol can be reflected in hair assessed after birth [46]. Although our study reflected that high maternal hair cortisol levels during postpartum can improve cognitive and motor development, our results reflect that high neonate hair cortisol levels during the first, second, and third trimester negatively affect their neurodevelopment. According to our findings, high neonate hair cortisol levels were related to a higher motor development in six-month-old infants. Our study disagrees with a previous study reporting that high neonate cortisol levels due to early neonatal treatment using a cortisol-derived substance (hydrocortisone) may have undesired effects on neurodevelopment in infants [47]. Nonetheless, a prior study reported no association between infants’ cortisol levels from blood samples with their neurodevelopment when assessing motor and cognitive development [48], probably since blood cortisol levels indicate acute stress, and hair cortisol reflects chronic stress [15]. Consistent with our study, those neonates exposed to high levels of cortisol during their intrauterine life showed a lower neurodevelopment [49,50]. Although hair cortisol has been previously used as a biological marker of chronic stress in neonates [51], our study is the first one to use hair cortisol levels from neonates to predict neurodevelopment in infants. Despite the fact that infants´ hair cortisol levels have been associated with maternal chronic stress during pregnancy in a previous study [39], no association was found between maternal and infant hair cortisol levels in the present study.

Strengths of this study include the longitudinal design that allowed assessing cortisol levels during the first, second, and third trimester, and postpartum in pregnant women, along with neonate cortisol levels. Additionally, the use of hair to assess cortisol levels through a non-invasive method provides a retrospective stress status of pregnant women during the periconception period and gives the opportunity to estimate fetal cortisol [42,43,46]. Moreover, infant neurodevelopment was assessed at the early age of six months which may allow the detection of potential associations between maternal and neonate hair cortisol levels and infant development at early stages. Previous studies assessing the association between maternal cortisol levels and infant neurodevelopment analyzed cortisol levels from blood, urine, or amniotic fluid samples [9,21,52].

A limitation of the present study comes from its longitudinal design; i.e., a large number of participants decided not to continue collaborating at different points in time. An additional potential limitation was that infant neurodevelopment was only assessed at a single point in time when infants were six months of age. Future studies may introduce in their protocols a long-term follow-up that may offer the possibility to assess the implications of prenatal and postnatal maternal and neonate cortisol levels on infant development. Moreover, maternal psychological variables and environmental influences of viruses that may be related with infant development were not assessed in our study [53,54]. Future studies should explore potential associations between pregnancy-specific stress and infant neurodevelopment. Pregnancy-specific stress informs of the levels of stress pregnant women have in respect to their medical health, delivery, relationships, and the possibility of having a premature baby [55,56]. Furthermore, studies investigating the association between maternal psychological stress or maternal cortisol levels can benefit if they include pregnant women using assisted reproductive technology (ART). A recent study has reported an association between maternal hair cortisol levels and infants´ development among pregnant women using ART [57]. More precisely, this study reported that hair cortisol levels during the first trimester among pregnant women using ART was associated with their neonatal head circumference [57].

## 5. Conclusions

Maternal stress during pregnancy as reflected by maternal hair cortisol levels has an impact on infants’ neurodevelopment at six months of age.

Our findings revealed that high maternal hair cortisol levels during the first and second trimester of pregnancy are associated with lower motor development. Nevertheless, this association is not always negative, depending on the time during pregnancy, cortisol levels samples, and the neurodevelopment assessment tool used [5]. Thus, elevated maternal hair cortisol levels in the first trimester were associated with higher cognitive development. During the postpartum period, higher maternal hair cortisol levels stimulated cognitive and motor development. Although stress levels have been considered to be associated with negative outcomes [3,4], certain levels of stress can also be beneficial for the developing fetus [58]. Thus, a previous study reported an association between mild to moderate levels of stress and an accelerated motor and cognitive development. These findings come from a study using psychological measures in low-risk pregnant women [58]. In this respect, future studies should include biological measures of stress (e.g., hair cortisol levels) and high-risk pregnant women.

Regarding neonate cortisol, the association is positive. Specifically, higher neonate hair cortisol levels at one month of age were associated with a higher motor development among infants aged six months. Hair cortisol levels are a retrospective biomarker of chronic stress, informing of the levels of the stress a person has been experiencing during the last three months [6,13,15]. Neonatal hair cortisol levels at one month reflect the level of stress the fetus was exposed to while in the womb during the last trimester. In line with this finding, a previous study has reported the beneficial role cortisol has on the development of lung function and the prevention of future impairment [39].

In line with our study, changes in the hypothalamic–pituitary–adrenal axis during the preconception period may have long-term consequences [6]. In this regard, antenatal effective stress assessments and interventions should be applied widely during women´s reproductive time spans to improve fetal and infant neurodevelopment [59]. The findings reported in this study are of high value for midwives and obstetricians when taking care of pregnant women, and researchers, who may use them to prevent negative outcomes and enhance adequate development among infants. The perinatal period is a highly vulnerable stage when it is paramount to provide the best maternity care [60].

## Figures and Tables

**Table 1:** Differences in sociodemographic, obstetrics, and hair cortisol levels between Group 1 (final sample) and Group 2 (participants not attending the neurodevelopment assessment appointment).

		Group 1 * (*n* = 41) *X* (*SD*)/N (%)	Group 2 * (*n* = 34) *X* (*SD*)/N (%)	Test **	*p*-Value
**Sociodemographic Variables**					
Age (years)		32.90 (4.15)	33.06 (3.72)	−1.12	0.21
Nationality	Spanish	35 (85.40%)	29 (85.30%)	0.01	0.99
	Immigrant	6 (14.60%)	5 (14.7%)		
Marital status	Single/divorced/widow	4 (9.80%)	6 (17.60%)	1.01	0.31
	Married/cohabitant	37 (90.20%)	28 (82.40%)		
Employment situation	Working	33 (80.50%)	28 (82.40%)	0.04	0.83
	Unemployed	8 (19.50%)	6 (17.60%)		
Salary	<1000 **€**	15 (36.60%)	12 (35.30%)	0.12	0.93
	1000–2000 **€**	6 (14.60%)	6 (17.6%)		
	>2000 **€**	20 (48.8%)	16 (47.1%)		
Level of education (years)		13.90 (2.09)	13.29 (2.55)	1.13	0.26
Sport	Yes	22 (53.7%)	13 (38.2%)	1.77	0.18
	No	19 (46.30%)	13 (61.80%)		
Hair aspect	Nature	19 (46.30%)	14 (41.20%)	0.75	0.38
	Dyed	22 (53.70%)	20 (58.80%)		
**Obstetric Information**					
Primiparous	Yes	19 (46.3%)	16 (47.1%)	0.01	0.95
	No	22 (53.70%)	18 (52.90%)		
Wanted pregnancy	Yes	36 (87.8%)	28 (82.4%)	0.44	0.51
	No	5 (12.2%)	6 (17.60%)	0.74	0.86
Previous miscarriages	Yes	16 (39.0%)	18 (52.90%)	1.45	0.23
	No	25 (61.0%)	16 (47.10%)		
Labor and birth	Eutocic	34 (82.90%)	30 (88.20%)	1.72	0.42
	Dystocic	5 (12.20%)	4 (11.8%)		
	C-section	2 (4.90%)	0 (0%)		
Pregnancy method	Spontaneous	36 (87.80%)	31 (91.20%)	0.22	0.63
	Assisted reproductive Technique	5 (12.2%)	3 (8.8%)		
Sex of the fetus	Female	22 (53.70%)	19 (55.90%)	0.04	0.84
	Male	19 (46.3%)	15 (44.1%)		
Infant birth weight (g)		3200 (377.05)	3299 (379.46)	−1.12	0.26
**Hair Cortisol Levels**					
Maternal hair cortisol levels (pg/mg)	1st trimester	303.52 (392.35)	394.68 (497.01)	−0.88	0.38
	2nd trimester	422.64 (712.78)	373.88 (514.97)	0.33	0.74
	3rd trimester	386.46 (338.93)	375.38 (569.65)	0.11	0.92
	Postpartum (1 month)	919.27 (1536.71)	629.28 (1088.28)	0.92	0.36
Infant hair cortisol levels (pg/mg)	Postpartum (1 month)	2747.45 (2209.54)	2359.72 (1400.36)	0.87	0.38

Note: * Group 1 = Participants’ infants were assessed using the Bayley Scale of Infant Development (BSID); Group 2 = Participants’ infants were not assessed with the BSID. ** *T*-test was used to quantitative variables and chi-square test to categorical variables.

**Table 2:** Pearson’s correlations between maternal and infant hair cortisol levels.

		Maternal Hair Cortisol Levels				Neonatal Hair Cortisol Levels	VIF
		T1	T2	T3	T4	T5	
Maternal hair cortisol levels	T1		0.57 *	0.13	0.23	0.03	1.22
	T2			0.55 *	0.24	−0.11	1.34
	T3				0.14	−0.01	1.25
	T4					−0.09	1.07
VIF			1.13	1.19	1.09	1.05	

Note: VIF—variance inflation factor; T1 = first trimester; T2 = second trimester; T3 = third trimester; T4 = postpartum. * *p* < 0.05.

**Table 3:** Hierarchical regressions using maternal hair cortisol levels as predictors of infant neurodevelopment.

	BSID Scales		Cognitive	Receptive Language	Expressive Language	Fine Motor	Gross Motor
Maternal hair cortisol levels	T1	*R* ^2^	0.23	0.12	0.19	0.05	0.24
		β	0.08	0.02	0.18	0.06	−0.18
		F	1.94	0.91	1.66	0.38	2.5
		*p*	0.57	0.89	0.17	0.85	0.05 *
	T2	*R* ^2^	0.23	0.12	0.15	0.05	0.23
		β	0.07	−0.04	0.03	−0.06	−0.15
		F	2.19	1.01	1.32	0.38	2.11
		*p*	0.62	0.43	0.27	0.85	0.05 *
	T3	*R* ^2^	0.19	0.14	0.17	0.06	0.24
		β	−0.13	−0.15	−0.12	−0.11	−0.19
		F	2.12	1.17	1.44	0.45	2.25
		*p*	0.09	0.34	0.23	0.81	0.07 *
	T4	*R* ^2^	0.31	0.12	0.20	0.04	0.25
		β	0.30	0.01	−0.21	−0.01	0.21
		F	3.26	0.98	1.74	0.35	2.33
		*p*	0.04 *	0.43	0.15	0.87	0.05 *

Note: The model is adjusted for potential covariates (maternal level of education, salary, maternal age, and infant birth weight). * *p* < 0.05; T1 = first trimester; T2 = second trimester; T3 = third trimester; T4 = postpartum.

**Table 4:** Hierarchical regressions using neonatal hair cortisol levels as predictors of infant neurodevelopment.

		Cognitive	Receptive Language	Expressive Language	Fine Motor	Gross Motor
Neonatal hair cortisol levels	*R* ^2^	0.26	0.21	0.16	0.04	0.28
	β	0.13	−0.32	−0.04	−0.04	−0.31
	F	2.31	1.91	1.33	0.35	2.81
	*p*	0.41	0.07	0.27	0.87	0.03 **

Note: The model is adjusted for potential covariates (maternal level of education, salary, maternal age, and infant birth weight). ** *p* < 0.02.

**Table 5:** Summary of the hierarchical regression models using maternal and neonatal hair cortisol levels as predictors of infant neurodevelopment at six months of age.

			BSID Scales			
		Cognitive	Receptive Language	Expressive Language	Fine Motor	Gross Motor
Maternal hair cortisol levels	T1	Not significant	Not significant	Not significant	Not significant	↓
	T2	Not significant	Not significant	Not significant	Not significant	↓
	T3	Not significant	Not significant	Not significant	Not significant	Not significant
	T4	↑	Not significant	Not significant	Not significant	↑
Neonatal hair cortisol levels		Not significant	Not significant	Not significant	Not significant	↑

Note: T1 = first trimester; T2 = second trimester; T3 = third trimester; T4 = postpartum. ↑: Positive association based on the β coefficient from the hierarchical regression model adjusted for covariates (maternal level of education, salary, and infant birth weight). ↓: Negative association based on the β coefficient from the hierarchical regression model adjusted for covariates (maternal level of education, salary, and infant birth weight).

## Supplementary Materials

The following are available online at https://www.mdpi.com/2077-0383/8/11/2015/s1, Table S1: Differences in hair cortisol levels and infants´ neurodevelopment between boys and girls.
