# Supplementary material for: Maternal and Neonatal Hair Cortisol Levels Are Associated with Infant Neurodevelopment at Six Months of Age
Source: J Clin Med. 2019 Nov 19;8(11):2015. doi: 10.3390/jcm8112015 (PMC6912650; doi:10.3390/jcm8112015)
Supplement: Supplementary file 1 [file jcm-08-02015-s001.pdf]

Table S1. Differences in hair cortisol levels and infants´ neurodevelopment between boys and girls

		**Boys** (n=19) *X*(*SD*)	**Girls** (n=22) *X*(*SD*)	**T-Test**	**P-value**
**Maternal hair cortisol levels**	T1	317.20	378.18	-0.59	0.55
	T2	494.33	322.75	-1.18	0.24
	T3	393.57	371.37	-0.21	0.83
	T4	481.45	1041.87	1.81	0.08
**Neonatal Hair cortisol levels**		2490.73	2638.80	0.33	0.73
**Neurodevelopment**	Cognitive	30.21	31.27	0.94	0.35
	Receptive language	11.11	10.95	-0.24	0.81
	Expresive language	9.37	9.27	-0.11	0.91
	Fine Motor	20.68	20.86	0.19	0.84
	Gross Motor	23.68	23.95	0.26	0.79
